# DRY–WET CYCLES INCREASE PESTICIDE RESIDUE RELEASE FROM SOIL

**DOI:** 10.1002/etc.1851

**Published:** 2012-07-11

**Authors:** Nicolai David Jablonowski, Andreas Linden, Stephan Köppchen, Björn Thiele, Diana Hofmann, Peter Burauel

**Affiliations:** † Institute of Bio- and GeosciencesIBG-3: Agrosphere, Forschungszentrum Jülich, Germany; ‡ Institute of Bio- and GeosciencesIBG-2: Plant Sciences, Forschungszentrum Jülich, Germany; § BioSpec, Forschungszentrum JülichGermany

**Keywords:** Pesticides, Persistence, Remobilization, Leaching, Risk assessment

## Abstract

Soil drying and rewetting may alter the release and availability of aged pesticide residues in soils. A laboratory experiment was conducted to evaluate the influence of soil drying and wetting on the release of pesticide residues. Soil containing environmentally long-term aged (9–17 years) ^14^C-labeled residues of the herbicides ethidimuron (ETD) and methabenzthiazuron (MBT) and the fungicide anilazine (ANI) showed a significantly higher release of ^14^C activity in water extracts of previously dried soil compared to constantly moistened soil throughout all samples (ETD: *p* < 0.1, MBT and ANI: *p* < 0.01). The extracted ^14^C activity accounted for 44% (ETD), 15% (MBT), and 20% (ANI) of total residual ^14^C activity in the samples after 20 successive dry–wet cycles, in contrast to 15% (ETD), 5% (MBT), and 6% (ANI) in extracts of constantly moistened soils. In the dry–wet soils, the dissolved organic carbon (DOC) content correlated with the measured ^14^C activity in the aqueous liquids and indicated a potential association of DOC with the pesticide molecules. Liquid chromatography MS/MS analyses of the water extracts of dry–wet soils revealed ETD and MBT in detectable amounts, accounting for 1.83 and 0.01%, respectively, of total applied water-extractable parent compound per soil layer. These findings demonstrate a potential remobilization of environmentally aged pesticide residue fractions from soils due to abiotic stresses such as wet–dry cycles. Environ. Toxicol. Chem. 2012; 31: 1941–1947. © 2012 SETAC

## INTRODUCTION

In agriculture, approximately 80% of all pesticides are used for crop protection, and between 1995 and 2007 pesticide use exceeded 5.0 billion pounds (2.3 million metric tons) annually worldwide 1–5. Due to the large quantities of pesticides applied annually to fields, the receiving surface soils function as sinks for a number of chemicals and as buffers for deeper soil layers and aquifers. Surface soils exposed to pesticide applications are also subject to various abiotic influences such as repeated and strong drying and wetting cycles and mechanical disruption such as tillage, which influence the fate and disposition of pesticides in soils. Therefore, the impact of changing climatic conditions is also a subject of growing concern with regard to the distribution of chemical pollutants in the environment 6.

Soil organic carbon (SOC) plays an important role in soil fertility and pesticide fate. The effect of soil drying and wetting on the release of SOC has been described elsewhere 7, mainly with regard to soil aggregation, soil aggregate stability 8–10, and decomposition of SOC 7, 8, 11–14. A decline in SOC is a matter of growing concern 15, and it is likely to be exacerbated by intensified agricultural production and changing climatic conditions 16, with potentially adverse consequences on soil fertility, soil stability, and food production. Soil organic carbon has a substantial influence on organic pesticide sorption and retention, which influence the fate of these substances in the environment. As a consequence, it can be assumed that a decrease in SOC might also result in increased mobilization of pesticide residues in soils due to missing binding sites. Soil disaggregation, promoted by dry–wet cycles, results in increased release of SOC 9, 10 and release of associated pesticide residues, as previously suggested 17. In turn, released SOC might be subject to increased microbial mineralization 12, 18. In addition, microbially degradable carbon sources in soil may promote the degradation of pesticides cometabolically.

**Fig. 1:**
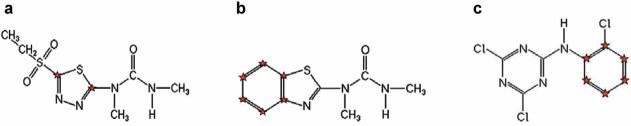
Molecular structure of (**a**) ethidimuron (ETD), (**b**) methabenzthiazurone (MBT), and (**c**) anilazine (ANI). 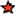 = ^14^C-labeling position. [Color figure can be seen in the online version of this article, available at http://wileyonlinelibrary.com.]

Experiments on the long-term fate of aged ^14^C-labeled pesticides in soils are rarely conducted. Outdoor lysimeter studies using ^14^C-labeled pesticides provide important information in this respect. The influence of dry–wet cycles of soil on the water extractability and release of long term–aged ^14^C-labeled pesticide residues has not been systematically tested and will provide important information about the remobilization of pesticide residues under changing abiotic conditions. Therefore, the present study investigated the influence of successive dry–wet cycles under laboratory conditions on the release of ^14^C-labeled residues of the thiadiazolylurea herbicide ethidimuron (ETD; 1-[5-ethylsulfonyl-1,3,4-thiadiazol-2-yl]-1,3-dimethylurea), the dimethylurea herbicide methabenzthiazuron (MBT; 1-[1,3-benzothiazol-2-yl]-1,3-dimethylurea), and the triazine fungicide anilazine (ANI; 4,6-dichloro-N-[2-chlorophenyl]-1,3,5-triazin-2-amine) (Fig. 1) in soils aged under environmental conditions for 9 to 17 years. The following aims were addressed: (1) assessment of the influence of successive soil dry–wet cycles compared to permanently moistened soil on the water extractability of the aged ^14^C-labeled pesticide residues, (2) quantitative and qualitative analyses of the pesticide residues in the soil water extracts, and (3) evaluation of the influence of dry–wet cycles on SOC and total nitrogen (TN) extractability.

## MATERIALS AND METHODS

### Soil and lysimeter history

All outdoor lysimeters had a surface area of 1 m^2^ and consisted of an undisturbed soil column (soil depth 1.1 m) of an Orthic Luvisol (C_org_: 1.2%, sand: 6.4%, silt: 78.2%, clay: 15.4%; pH: 7.2) 19–21. The time and amount of ^14^C-pesticide applications on the lysimeter soils are presented in Table 1. The chemical properties of the parent pesticide compounds are summarized in Table 2.

**Table 1:** Application time and total quantities of applied ^14^C-labeled and nonlabeled ethidimuron (ETD), methabenzthiazuron (MBT), and anilazine (ANI) on lysimeter soils

Soil	Date of application (dd/mm/yyyy)	Applied a.i.^a^ (mg m^−2^)	Total applied a.i. (kg ha^−1^)	Applied 14C activity (MBq m^−2^)	Specific ^14^C activity (kBq mg^−1^)
ETD soil	13.11.1997	123.3	1.23	107.3	870.0
MBT soil	29.11.1994 (last application)	879.0^b^	8.79^b^	261.6^b^	297.7
ANI soil	13.06.1989 (last application)	1978^c^	19.78^c^	372.5^c^	188.4

a a.i. = active ingredient.

b In total after three applications, 1988, 1992, and 1994.

c In total, after five consecutive annual applications, 1985-1989. Sampling of all lysimeter soils was performed in April 2006.

**Table 2:** Chemical properties of ethidimuron (ETD), methabenzthiazuron (MBT), and anilazine (ANI)^a^

Pesticide	Molecular formula	Chemical family	Molecular weight (g Mol^−1^)	Solubility in water (g L^−1^ at 20°C)
ETD	C_7_ H_12_N_4_O_3_S_2_	Urea; thiadiazole	264.33	3.0
MBT	C_10_H_11_N_3_OS	Urea; benzothiazole	221.29	0.059
ANI	C_9_H_5_C_l3_N_4_	Triazine; organochlorine	275.54	0.008

a Information taken from *The Agrochemicals Handbook* 22.

### Soil sampling and ^14^C-residue detection

A total of approximately 2 kg of each individual lysimeter soil was sampled randomly in April 2006 from the soil layer at 0 to 30 cm depth of the ETD, MBT, and ANI lysimeter, using a Humax stainless steel soil core sampler, 3 cm in diameter. Because no ploughing simulation was applied on the ETD soil within six years prior to sampling as was performed for MBT and ANI soils, ETD soil was subdivided into 10-cm layers and only the soil from 0 to 10 cm depth was used for this experiment. For further ^14^C-residue calculation, estimated bulk soil densities of 1.5 g cm^−3^ for ETD soil and 1.3 g cm^−3^ for MBT and ANI soil were assumed. Soils were air-dried to a residual moisture content of 7 to 12% and then sieved (2 mm), homogenized, and stored in the dark at 3°C. To detect residual ^14^C activity in soils, samples of 50 g were dried at 105°C to accelerate the experimental process and ground in a mortar. Volatilization of pesticide residues during the drying process at 105°C can be excluded because results were compared with those obtained from soil samples that were air-dried only and found to be not different. The very low vapor pressure for these compounds (ETD: < 0.001 mPa; MBT: ≍590 nPa; ANI: 910 nPa; all at 20°C 22) supports this observation. Subsamples were oxidized in five parallels of 1 g (Biological Oxidizer OX500; R.J. Harvey Instrument). Evolving ^14^CO_2_ was trapped (Oxysolve C-400 scintillation cocktail; Zinser Analytik), and radioactivity was quantified using a liquid scintillation counter with internal quench correction (2500 TR, Tri-Carb; Packard Liquid Scintillation Analyzer).

### Soil organic carbon and nitrogen analyses in soil samples

The SOC and TN were determined in triplicates of freeze-dried and homogenized soil samples prior to water-shaking extractions, as described previously 23. Organic C was determined by radiofrequency heating of a 100-mg sample in flowing oxygen and subsequent infrared absorption by a Leco RC-412 multiphase carbon determinator. A Leco TCH 600 was applied to determine N_2_ by thermal conductivity detection using 2-mg samples.

### Desorption experiments

For each soil, two parallel experiments (A and B) were established. Triplicates of 10 g for each soil containing the aged ^14^C-labeled and nonlabeled ETD, MBT, and ANI residues were either (A) directly mixed with distilled water (1 + 2, w:w) or (B) first oven-dried at 45°C until dryness before adding water (1 + 2, w:w). All samples were simultaneously shaken for 1 h at 150 rpm on a horizontal shaker at room temperature (21 ± 2°C) and subsequently centrifuged to separate the solids from the water phase (60 min, 3,000 rpm, equal to approximately 2,800 *g*; Allegra 6KR, GH-3.7 Horizontal Rotor; Beckmann Coulter). The resulting supernatants were filtered (0.45 µm, Porafil MV; Macherey-Nagel) to remove potential particulates from all water-extract samples prior to the detection of dissolved ^14^C activity, dissolved organic carbon (DOC), dissolved TN, pH, and electrical conductivity. For setup A, all moistened soil samples were stored after centrifugation and removal of the supernatant in the dark at 3°C, until all samples of setup B were again completely dried at 45°C (approximate drying time 3–4 d), and the cycle could be repeated. Both setups A and B were subject to 20 successive water-shaking extractions.

Subsequent to the entire experiment, total ^14^C recovery was calculated for all individual soils as a sum of total ^14^C activity extracted from each soil sample and the residual ^14^C activity remaining in the water-extracted soil samples, as well as ^14^C activity adsorbed to the filters.

### Determination of water-extracted ^14^C activity

The amount of dissolved ^14^C activity was determined in triplicates for each soil extract by mixing 1 ml of the liquid samples with 10 ml of scintillation cocktail (Instant Scint-Gel Plus; PerkinElmer), and radioactivity was detected by liquid scintillation counting. An external standard was used for quenching correction using 1 ml of distilled water with 10 ml of scintillation cocktail.

### Carbon and nitrogen analysis of water extracts

All aqueous samples were analyzed for total DOC and dissolved TN content, using a Shimadzu Total Organic Carbon/Nitrogen Analyzer (TOC-5050A, ASI-5000A Auto Sampler).

### Liquid chromatography MS/MS analysis of water extracts

#### Reagents and materials

Bayer Crop ScienceAG provided ETD, MBT, ANI, dihydroxyanilazine (di-OH-anilazine), and dimethoxyanilazine (di-OMe-anilazine). Isoproturone (IPU) and d_5_-2-hydroxyatrazine (purchased from Dr. Ehrenstorfer GmbH) were used as internal standards for quantification of ETD, MBT, and ANI and of di-OH-anilazine and di-OMe-anilazine, respectively. Ammonium acetate (Fractopur), formic acid (100%, Suprapur), and acetonitrile (LiChrosolv) were obtained from Merck. Water obtained from a Milli-Q-Plus Ultra-Pure water purification system (Millipore) was used to prepare all samples and mobile phases.

#### Liquid chromatography atmospheric pressure chemical ionization–tandem mass spectrometry analysis

Liquid chromatography–atmospheric pressure chemical ionization–tandem mass spectrometry analysis (LC-APCI-MS/MS) was applied in accordance with our method described elsewhere 24. Briefly, an Agilent 1100 series HPLC coupled with a Thermo Electron TSQ Quantum triple quadrupole mass spectrometer was used. Liquid chromatographic separations were carried out with a Phenomenex Synergi 4µ Polar RP18 column, 150 × 3.0 mm I.D. A 1 mM ammonium acetate + 0.1% formic acid (A)/acetonitrile + 0.1% formic acid (B) gradient was applied for ETD and MBT, while ANI and ANI metabolites were separated with 5 mM ammonium acetate (A) and acetonitrile (B). The mass spectrometer was operated in the positive atmospheric pressure chemical ionization (APCI(+)) mode for the detection of ETD and MBT and in the negative atmospheric pressure chemical ionization (APCI(−)) mode in case of ANI and ANI metabolites. Multiple reaction monitoring was used to quantify all analytes and standards.

#### Calibration and quantification

Quantification was carried out using the internal standard method as stated previously 24. Analysis of MBT/ETD was performed with IPU as an internal standard and that of ANI and ANI metabolites with d_5_-2-hydroxyatrazine.

### Electrical conductivity and pH analyses of water extracts

To determine the potential loss of soil minerals as a result of the successive dry–wet cycles, the electrical conductivity of previously filtered water extracts was measured using a WTW device (Cond 340i/SET, WTW). The pH of all filtered water extracts was monitored using a Metler Toledo pH meter (PTB01 ATEX 2166X, Type 1120X).

### Statistical analysis

For statistical analysis, the independent two-sample *t* test was applied to determine the significance of differences between mean values.

## RESULTS AND DISCUSSION

### Soil analyses

Even after long-term environmental aging (9–17 years) (Table 1), a major fraction of residual ^14^C activity was still present in the upper soil layer of 0 to 10 cm depth for ETD soil and 0 to 30 cm depth for MBT and ANI soil, accounting for 18.7% (ETD soil), 34.8% (MBT soil), and 43.2% (ANI soil) of total initially applied ^14^C activity (Table 3). Considering additionally the detected residual ^14^C activity in the ETD soil layers at 10 to 20 and 20 to 30 cm depths, the residual detected ^14^C activity accounted for 16.4 and 12.2% (data not shown), respectively. In this case, the total residual ^14^C activity accounted for 47.3% of initially applied ^14^C activity in the entire ETD soil layer at a depth of 0 to 30 cm. As presented in another environmental long-term study using ^14^C-labeled atrazine 23, 25, 26, most of the residual ^14^C activity was located in the upper 10-cm soil layer and is most likely associated with the higher organic carbon content in this soil layer 26–28.

**Table 3:** Total residual ^14^C activity per soil layer and total water-extracted ^14^C activity after 20 consecutive water extraction steps

Soil (layer in cm)	Residual ^14^C activity of total applied in soil layer (%)^a^	Total water-extractable ^14^C activity in sample (%)	Total water-extractable parent compound (µg kg^−1^)	Parent compound of initially applied calculated for m^2^ and soil layer (%)^a^
ETD (0–10)	18.71	Dry–wet: 43.8 ± 5.5	15.1 ± 3.7	1.83
		Wet: 15.5 ± 1.7	2.8 ± 1.2	0.34
MBT (0–30)	34.84	Dry–wet: 15.3 ± 0.9	0.23 ± 0.06	0.01
		Wet: 4.6 ± 0.9	—	—
ANI (0–30)	43.24	Dry–wet: 19.5 ± 1.6	—	—
		Wet: 6.1 ± 0.4	—	—

a Estimated soil density for calculation: Ethidimuron (ETD) soil 1.5 g cm^−3^; methabenzthiazuron (MBT) / anilazine (ANI) soil 1.3 g cm^−3^; ± standard deviation of *n* = 3 to 9.

The SOC and TN contents in the soils at the date of sampling accounted for 1.14 ± 0.06% and 0.15 ± 0.00%, respectively. Because all soil monoliths were taken at the same field plot, minor differences in SOC content can be attributed to potential differences in the soil management of the lysimeters 24.

An association of ETD, MBT, and ANI with SOC has previously been demonstrated 21, 29. Even after long-term environmental aging, SOC plays a major role in pesticide residue retention in the upper soil layers, where the SOC content is generally higher than in deeper layers.

A decrease in SOC content could be related to intensified agricultural production and climatic changes leading to a potential increase of SOC microbial turnover; however, current scientific data on SOC decline in soils are rather inconsistent with regard to this issue 30–34. A decrease in SOC could be facilitated by soil drying and rewetting due to a subsequent increase in microbial SOC turnover 35. Reemtsma et al. 17 suggested that the contaminant release from soils might be influenced by microbial activity over time, altering the long-term function of soils as a sink or source of organic contaminants.

Considering a potential decline in SOC in agricultural soils, it can be assumed that the buffering function of the soils for pesticides and their metabolites could be reduced. This might result in greater remobilization of pesticides in soils due to altered SOC content.

### Analyses of water extracts

#### Liquid chromatography MS/MS analyses

**Fig. 2:**
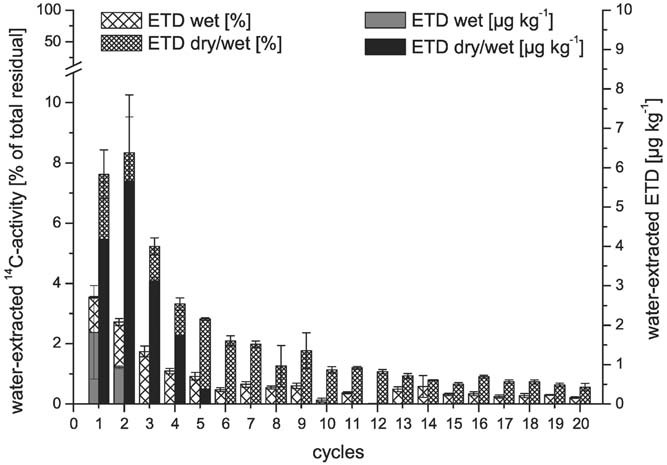
Water-extracted ^14^C activity (left *y* axis, percentage of total residual ^14^C activity) and liquid chromatography MS/MS-detected compound (right *y* axis, in µg kg^−1^ soil) in water extracts of ethidimuron (ETD) soil. Values for ^14^C activity and the detected parent compound were significantly higher in dry–wet versus constantly moistened soil water extracts (*p* < 0.001, except cycle 20: *p* < 0.01 and cycles 8 and 14: *p* < 0.1). Standard deviation *n* = 9.

**Fig. 3:**
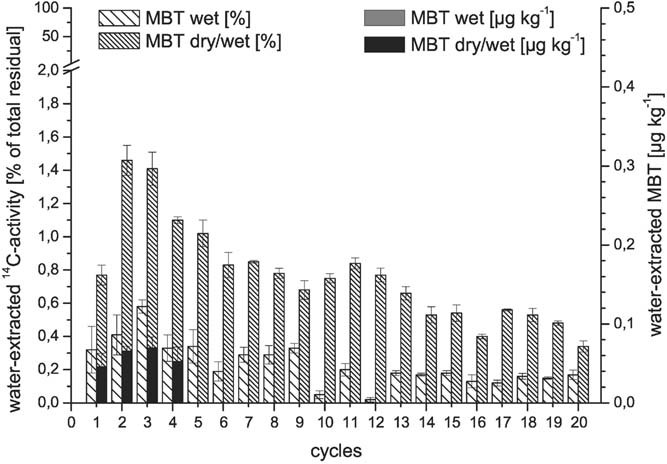
Water-extracted ^14^C activity (left *y* axis, percentage of total residual ^14^C activity) and liquid chromatography-MS/MS-detected compound (right *y* axis, in µg kg^−1^soil) in water extracts of MBT soil. Values for ^14^C activity and the detected parent compound were significantly higher in dry–wet versus constantly moistened soil water extracts (*p* < 0.001, except cycle 1: *p* < 0.01). Standard deviation *n* = 9.

Even after long-term environmental aging, liquid chromatography MS/MS analyses revealed the parent compounds ETD (nine years after application, Table 1) and MBT (12 years after application, Table 1) in water extracts of dry–wet soils, as presented in Figures 2 and 3, right *y* axis. The parent compound ETD was found in the first five water extracts of dry–wet soils and in the first two water extracts of constantly moistened soils, albeit in much smaller quantities (Fig. 2, right *y* axis), totaling 15.1 and 2.8 µg kg^−1^ of the water-extractable parent compound ETD, respectively (Table 3). As shown in Table 3, these values correspond to 1.83 and 0.34%, respectively, of the initially applied parent compound.

In the case of MBT, the parent compound was detected in the first four water extracts of dry–wet soil samples, totaling 0.23 µg kg^−1^ water-extractable parent compound MBT (Table 3). No MBT was detected in water extracts of constantly moistened soils (Fig. 3, right *y* axis). The detected amount of water-extractable MBT equals 0.01% of the initially applied parent compound.

This observation must be attributed to a combination of a generally higher water solubility of ETD (Table 2; 3.0 g ETD vs 0.059 g MBT L^−1^ at 20°C), a low adsorption affinity to soils, as well as a much higher environmental persistence in soils compared to MBT 22, 24, 36, 37. Although ETD is much more water-soluble, its biodegradability is much lower than that of MBT, resulting in proposed environmental half-lives of 162 to 2,059 d compared to approximately 24 to 230 d for MBT 21, 37, 38. Furthermore, MBT was found to be biodegradable 38–40, giving an additional explanation for the overall smaller MBT residue fractions in the water extracts. In contrast, the microbial ETD degradation in soil was found to be very limited 21.

In the case of ANI, neither the parent compound nor dihydroxy-ANI as its main metabolite was detected in the water extracts. This fact must be attributed to the very low water solubility of ANI (Table 2; 0.008 g L^−1^ at 20°C), its high adsorption affinity to the soil matrix, and the extended environmental aging time of 17 years (Table 1) as a driving factor for the formation of soil-bound residues 22, 29, 41, 42.

As shown in a previous study, drying and wetting soils containing phenanthrene and di(2-ethylhexyl)-phthalate reduced the biodegradability, extractability, and uptake by earthworms 43. In our case, however, the parent pesticide compounds ETD and MBT were detected in the water phase after long-term aging and thus could be more accessible for biodegradation or uptake. Generally, the increased release of aged pesticide residues from dried and rewetted soils could be due to disruption of soil aggregates, facilitating the release of entrapped pesticide molecules, which are normally excluded from remobilization under moistened conditions. These observations might also be valid for a number of other pesticide residues in soils, which could be remobilized by environmentally relevant dry–wet cycles. These data help to assess the chemical nature of long-term aged pesticide residues in soils and their remobilization potential. Biological and toxicological effects such as endocrine disrupting activity, as discussed for small concentrations of atrazine 44, are so far not published for the pesticide compounds described herein. Therefore, further research is needed to evaluate the biological or toxicological relevance of our findings.

#### Water-extracted residual ^14^C activity and DOC/TN determination

**Fig. 4:**
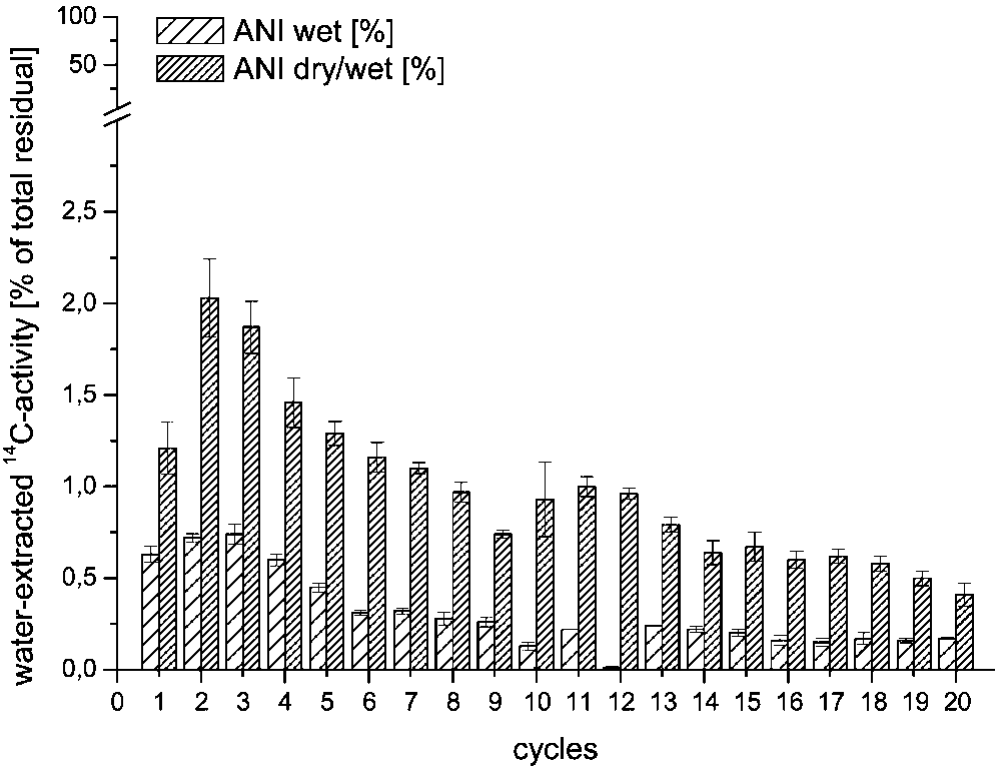
Water-extracted ^14^C activity (percentage of total residual ^14^C activity) in water extracts of anilazine (ANI) soil. Values for ^14^C activity were significantly higher in dry–wet versus constantly moistened soil water extracts (*p* < 0.001, except cycles 1 and 10: *p* < 0.01). Standard deviation *n* = 9.

The results showed a significant increase in desorbed ^14^C activity in water extracts of all soils after drying, accounting for 44% (ETD), 15% (MBT), and 20% (ANI) of residual ^14^C activity in soil samples after 20 dry–wet cycles compared to water extracts of constantly moistened soils (Figs. 2–4, left *y* axis). The amounts of water-extracted ^14^C activity from constantly moistened soil remained significantly lower at 16% (ETD), 5% (MBT), and 6% (ANI) of residual ^14^C activity in soil samples after 20 cycles. This observation clearly indicates that release of residual pesticide ^14^C activity is strongly influenced by soil drying and rewetting, irrespective of the chemical pesticide class. However, the water-extractable amount of residual pesticide ^14^C activity, and therefore the pesticide or its metabolites, is variable, depending on the specific water solubility of the individual compound.

**Fig. 5:**
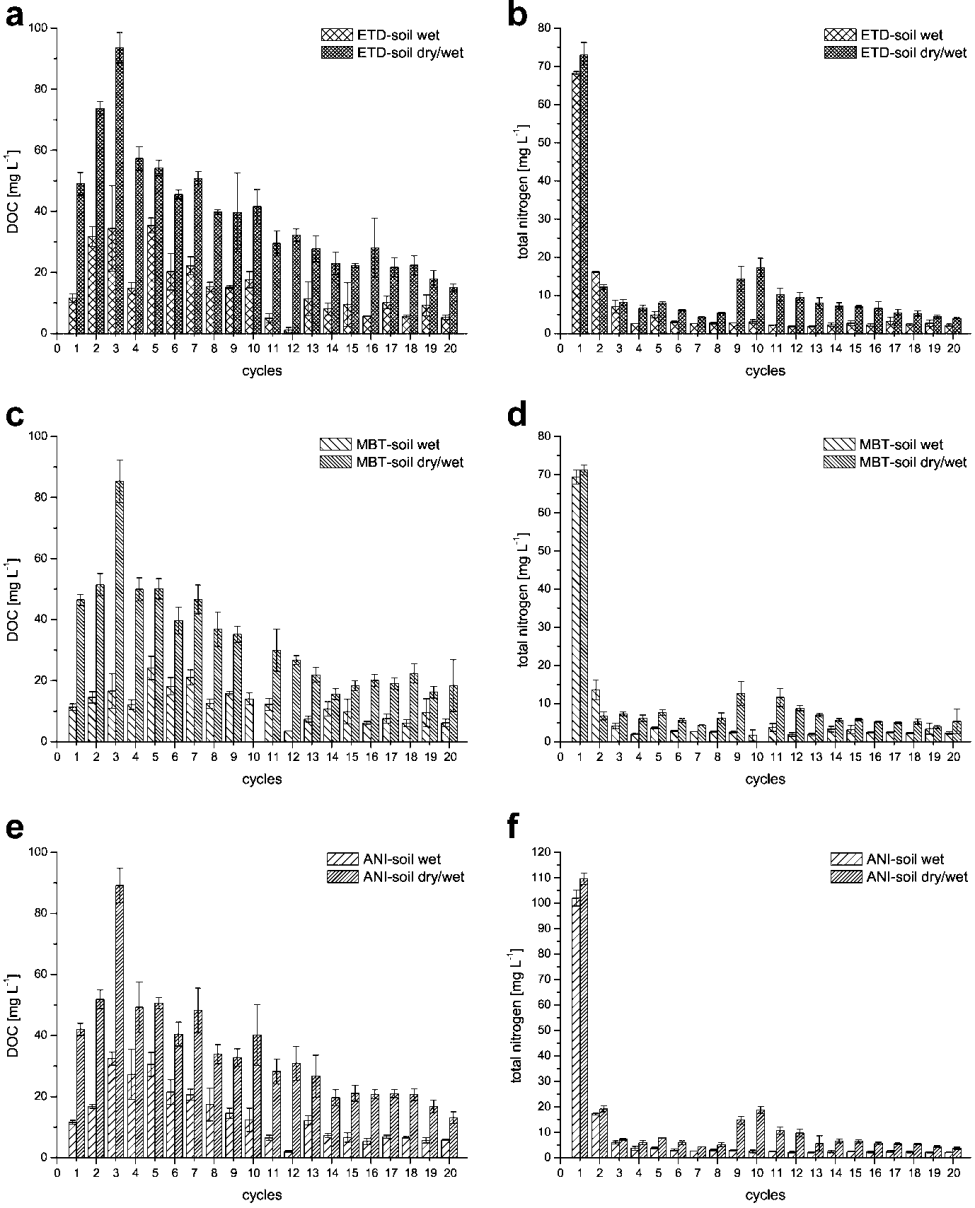
Dissolved organic carbon (DOC) and total nitrogen (TN), in µg L^−1^ water extract, of (**a, b**) ethidimuron (ETD) soil, (**c, d**) methabenzthiazurone (MBT) soil, and (**e, f**) anilazine (ANI) soil. Values for DOC and TN were significantly higher in dry–wet versus constantly moistened soil water extracts ([**a**]: *p* < 0.001–0.02, except cycle 15: *p* < 0.1; [**b**] *p* < 0.001–0.02, except cycles 1 and 17: *p* < 0.1; [**c**] *p* < 0.001–0.01, except cycles 14, 15, 19, 20: *p* < 0.1; [**d**] *p* < 0.001–0.02, except cycles 1, 19, 20: *p* < 0.1; [**e**] *p* < 0.001–0.02; [**f**] *p* < 0.001–0.02, except cycles 2, 3, 13: *p* < 0.1). Standard deviation *n* = 9.

As shown in Figure 5a, c, and e, the DOC content was significantly higher in all water extracts obtained from soils exposed to dry–wet cycles compared to those from constantly moistened soils. This is in accordance with previous observations 7. After 20 water-extraction cycles, the DOC content accounted for 11 ± 1% versus 5 ± 0.3% (ETD soil), 8 ± 0.4% versus 4 ± 0.3% (MBT soil), and 10 ± 1% versus 5 ± 0.2% (ANI soil) of the total organic soil carbon detected as DOC in dry–wet versus constantly moistened soil water extracts. The DOC content correlated positively with the measured ^14^C activity in the aqueous liquids, and the correlation was more pronounced in dry–wet soil water extracts than in water extracts of constantly moistened soils (*r* = 0.80–0.91 vs *r* = 0.41–0.70).

The overall observation of the increased water extractability of residual pesticide ^14^C activity and DOC from dry–wet soils compared to constantly moistened soils must be attributed to a physical disruption of soil aggregates, hosting residual pesticide molecules and potentially associated DOC in micropores. A physical disruption of soil aggregates due to dry–wet cycles resulting in extended release of DOC has also been discussed previously 7, 8. Throughout 20 successive dry–wet cycles, continuous disaggregation of soil particles resulted in higher release of residual pesticide ^14^C activity and DOC. Each dry–wet cycle changed the stability of aggregates. Air-dried aggregates are stable, and an increase in water potential decreases the stability, causing a new equilibrium to be established 10. This new equilibrium generally results in a reduced amount of protected SOC and an increased amount of rather unprotected DOC. Residues of organic pollutants are part of this cycling.

At this point, the binding mechanisms and the nature of DOC-associated pesticide residues remain unknown and need further investigation. However, the wet–dry cycles induced the release of SOC. As such, the pesticides went into solution as well, where they might be subject to degradation.

The release of TN was highly pronounced in the first extracts of both dry–wet and constantly moistened soil water extracts in all cases (Fig. 5b,d,f). It is likely that water-dissolvable inorganic nitrogen salts, such as potassium and sodium nitrates, were dissolved preferentially in the first water extracts. Throughout 20 successive dry–wet cycles, the measured TN content remained significantly higher in dry–wet versus constantly moistened soil water extracts. In accordance with the DOC contents in dry–wet soil water extracts, it can also be assumed that the higher TN values in these soil water extracts was related to increased mobilization of organic nitrogen fractions. This assumption can be supported by the small but relatively higher correlation between the TN and DOC contents in dry–wet soil water extracts compared to water extracts of constantly moistened soils for all samples (*r* = 0.14–0.23 vs *r* = −0.07–0.05).

#### Electrical conductivity and pH

The differences between the electrical conductivity in dry–wet and constantly moistened soil water extracts were small but statistically significant (*p* = 0.01–0.1).

The highest values were obtained in the first water extracts, accounting for 656 µS cm^−1^ in dry–wet versus 613 µS cm^−1^ in moist soil water extract for ETD soil, 625 versus 632 µS cm^−1^ for MBT soil, and 917 versus 913 µS cm^−1^ for ANI soil. In the second extract obtained after water-shaking extraction, electrical conductivity decreased significantly by a factor of four to five in all samples, leveling off to 20 to 38 µS cm^−1^ in water extracts of constantly moistened soils versus 40 to 45 µS cm^−1^ in dry–wet water extracts. This finding indicates that dry–wet cycles in soils moderately trigger the release of ions and micronutrients. The pH value remained constant for all water extracts, with an average of 6.6 ± 0.3, indicating that successive dry–wet or wet–wet cycles had no influence on the pH value in water extracts.

The overall results demonstrate long-term binding and sequestration mechanisms of organic pesticides in soils. The data suggest that environmentally relevant dry–wet cycles may change interfacial soil properties intensively. This may result in increased remobilization and release of aged pesticides or pesticide metabolites in surface soils. However, the experimental setup was designed to describe a maximum possible remobilization potential of the aged residual pesticide fractions present in our experimental soils.
